# Thermal Behavior of *n*-Octanol and Related Ether Alcohols

**DOI:** 10.1021/acs.jced.4c00525

**Published:** 2024-11-20

**Authors:** Markus M. Hoffmann, Torsten Gutmann, Gerd Buntkowsky

**Affiliations:** † Department of Chemistry and Biochemistry, State University of New York Brockport, Brockport, New York 14420, United States; ‡ Institute of Physical Chemistry, Technical University Darmstadt, Peter-Grünberg-Straße 8, Darmstadt D-64287, Germany

## Abstract

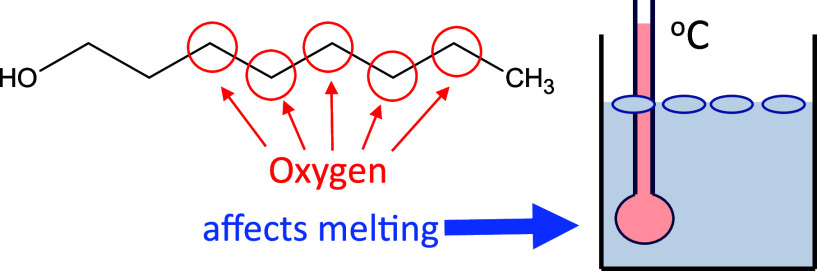

The thermal behavior of *n*-octanol and related ether alcohols has been studied by differential scanning calorimetry (DSC). The melting point, heat of fusion, and isobaric heat capacities of *n*-octanol obtained from the DSC measurements are in good agreement with literature values. The ether alcohols display kinetic barriers for forming a solid phase during cooldown. These barriers are least for 6-methoxyhexanol that forms a solid upon cooling except for the highest measured temperature change rate of 40 K·min^–1^, followed by 4-propoxybutanol that forms a solid during cooldown only at low cooling rates. 2-Pentoxyethanol and 5-ethoxypentanol form a solid during the heating cycle that then melts again upon further heating. 3-Butoxypropanol does not display any exo- and endothermic features for all measured temperature change rates. Consequently, new data on melting point and heats of fusion are reported for the ether alcohols except for 3-butoxypropanol. New isobaric heat capacities are presented as well for the liquid phase of these ether alcohols.

## 1 Introduction

In a recent study,^1^ we reported new experimental data on density, viscosity, and self-diffusion coefficients of *n*-octanol and the related ether alcohols shown in Table 1. The intent of this recent study was to better understand the interplay between inter- and intramolecular hydrogen bonding for these molecules. While *n*-octanol can only engage in intermolecular hydrogen bonding via the hydroxy group, the introduction of an ether functionality present in the related ether alcohols in Table 1 allows for two additional hydrogen bonding interactions, namely inter- and intramolecular hydrogen bonding between the hydroxy group and the ether functionality. Indeed, these additional hydrogen bonding interactions were observed to cause in comparison to *n*-octanol increased densities, reduced viscosities and faster self-diffusion coefficients. The derived new understanding of the correlations between molecular structure and hydrogen bonding as well as the resulting macroscopic physical properties can then ultimately be applied to molecules with more complicated hydrogen bonding interactions such as polyethylene glycol (PEG), which possess multiple ether functionalities in addition to two hydroxy groups. PEG is an inexpensive environmentally benign chemical that has been successfully used as a solvent for chemical synthesis.^2−4^ A better understanding of PEG as a solvent would be very beneficial to further advance these green chemistry efforts.^5^ Unraveling the inter- and intramolecular hydrogen bonding interactions in PEG is likely to be the key to such understanding.

**Table 1:**
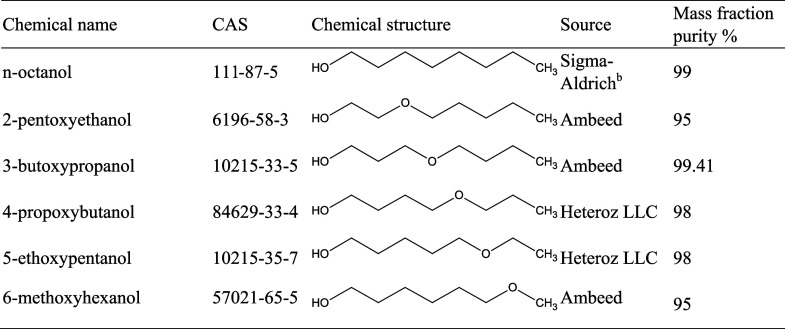
Information on Chemicals Studied

From a data availability point of view, we noticed that the ether alcohols in Table 1 are essentially unexplored with respect to their thermal behavior. We could only find in the literature an unreasonably high melting point of 110–111 °C reported for 3-butoxypropanol^6^ and a report on 2-penthoxyethanol stating that its phase behavior was observed to be vitreous.^7^ Therefore, we decided to study their liquid–solid phase behavior by DSC measurements. Included in this study is *n*-octanol as a reference compound to ensure accuracy of measurements of melting point temperature,^8^ heat of fusion,^9−11^ and temperature dependent isobaric molar heat capacities.^10,12−21^ We note that *n*-octanol is an amphiphile molecule used as a model molecule in studies of nonionic surfactants such as, for example, to mimic membranes.^22,23^ It is also used to assess the polarity and or hydro/lipophilicity of solute molecules through determination of their distribution coefficients between *n*-octanol and water.^24^

As we will show, the ether alcohols display vastly different thermal behavior compared to *n*-octanol. The presence of the ether functionality tends to hinder the formation of a crystalline solid to varying degrees in strong dependency on the position of the ether functionality in the ether alcohol structure. These barriers to the formation of a solid phase appear to be of kinetic nature as freezing processes are observed during the heating cycle before the sample remelts upon further heating. Despite these interfering processes new melting point temperatures as well as heat of fusion values and isobaric molar heat capacity data are extracted from the measurements and reported here.

## 2 Experimental Section

### 2.1 Preparation of Samples

Specifications of the investigated chemicals are listed in Table 1. No further purification was attempted. The chemicals were stored and samples were prepared in a glovebox (MBraun LabStar Eco) under nitrogen gas. Standard aluminum crucibles were filled with about 10 μL of sample and then press sealed. Sample mass determinations were obtained with an electronic balance of 0.01 mg precision (Satorius CPA 225D).

### 2.2 Differential Scanning Calorimetry (DSC)

A 214 Polyma NETZSCH DSC instrument was used to inspect the phase behavior of the samples and measure their specific heats as well as heats of fusion of observed phase transitions. Cooling was achieved by use of liquid nitrogen. The temperature reading of the instrument was calibrated using a set of six standards provided by NETZSCH. The correctness of the calibration for the temperature range relevant in this study was checked against the liquid-to-solid phase transition of *n*-octanol.^8^ For specific heat determinations sapphire crystals of four different thicknesses were used as standards to calibrate the heat flow reported by the instrument.^25^ The estimated standard uncertainties of melting point temperature, heat of fusion and isobaric molar heat capacities are discussed in Section 3.

## 3 Results and Discussion

### 3.1 Melting Points

**Figure 1:**
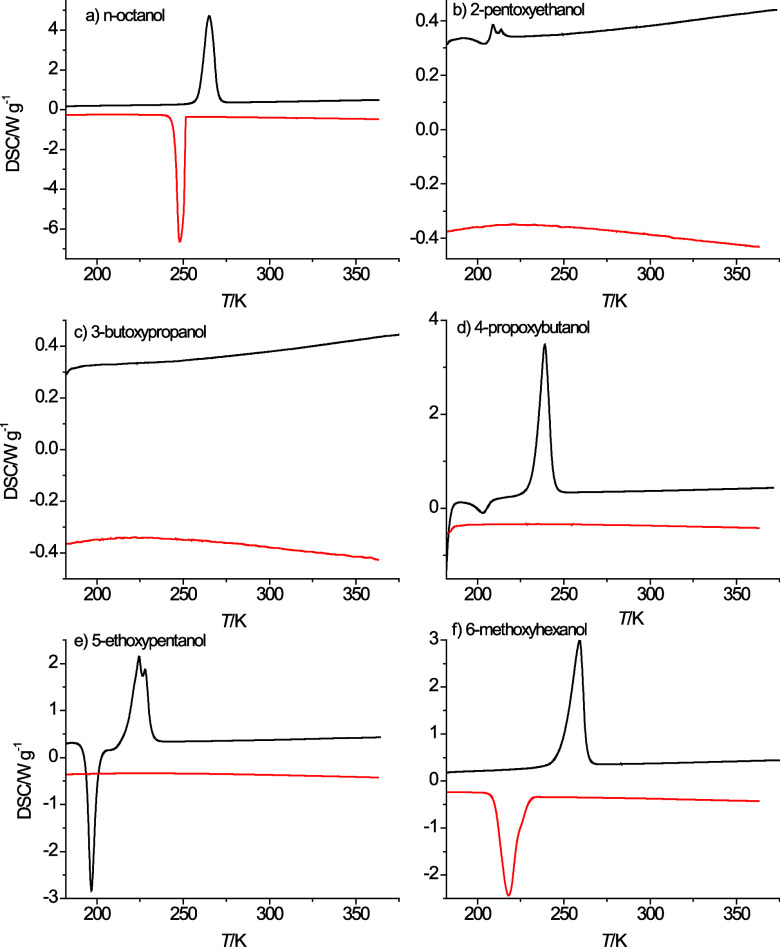
Cooling curves (red) and heating curves (black) obtained for *n*-octanol (a), 2-pentoxyethanol (b), 3-butoxypropanol (c), 4-propoxybutanol (d), 5-ethoxypentanol (e), and 6-methoxyhexanol (f) at a temperature change rate of 10 K·min^–1^.

**Figure 2:**
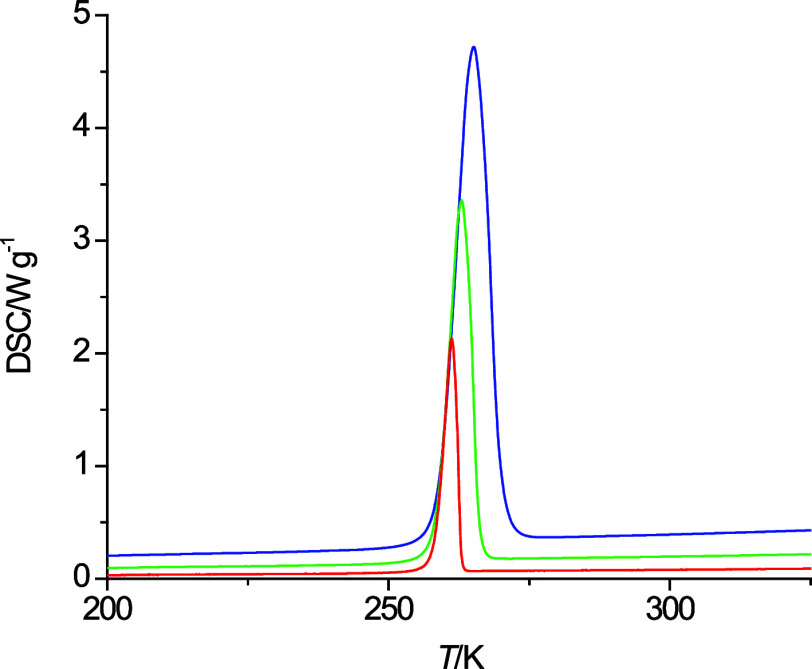
Heating curves of *n*-octanol at 10 K·min^–1^ (dark blue), 5 K·min^–1^ (green), and 2 K·min^–1^ (red).

Figure 1 shows the DSC cooling and heating curves at 10 K·min^–1^ obtained from the six alcohols listed in Table 1. The melting point of *n*-octanol is well established in the literature as 257 K.^8^ Hence, in Figure 1a an exothermic peak is observed just below 250 K for the liquid to solid phase transition and an endothermic peak is observed near 265 K for the solid-to-liquid phase transition. Typically, the onset temperature for the endothermic peak in the heating curve is taken as the melting temperature. The onset temperature is, however, temperature change rate dependent in DSC measurements.^26^ Hence, the DSC measurements for *n*-octanol were repeated at different rates of 5 K·min^–1^ and 2 K·min^–1^ (Figure 2) and the onset temperature extrapolated to a zero rate. The obtained temperature of 258 K is in agreement with the melting point of 257 ± 1 K listed in the NIST Chemistry WebBook.^8^

**Figure 3:**
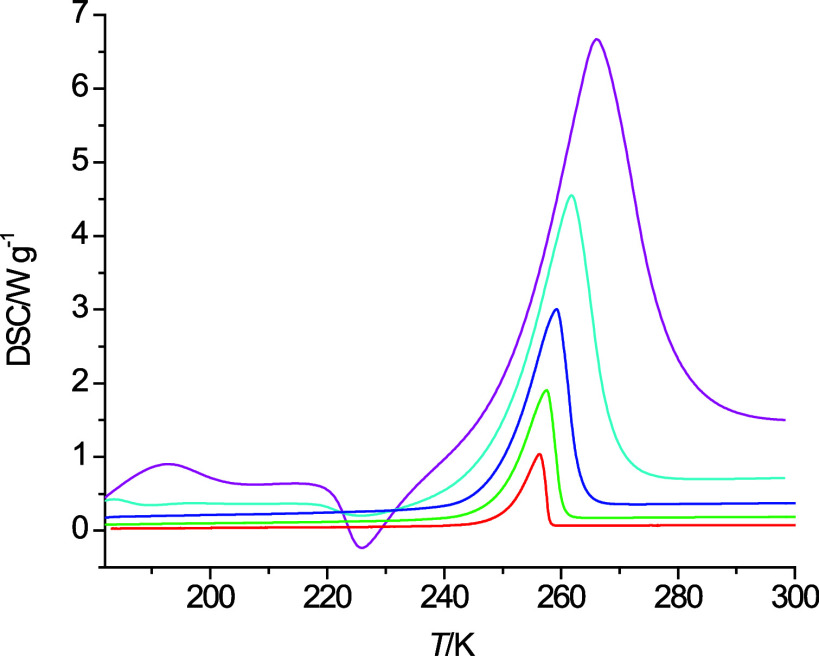
Heating curves of 6-methoxyhexanol at 40 K·min^–1^ (pink), 20 K·min^–1^ (light blue), 10 K·min^–1^ (dark blue), 5 K·min^–1^ (green), and 2 K·min^–1^ (red).

**Figure 4:**
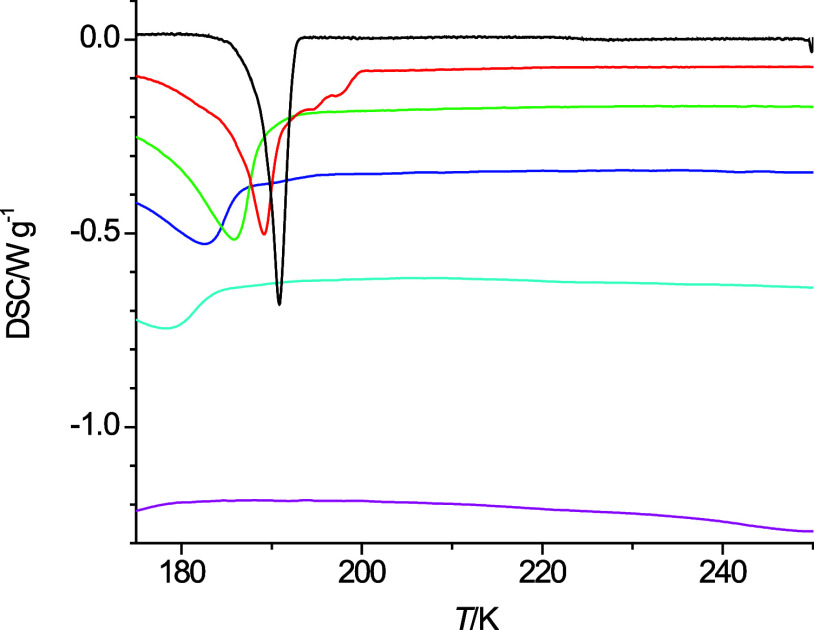
Cooling curves of 4-propoxybutanol at 40 K·min^–1^ (pink), 20 K·min^–1^ (light blue), 10 K·min^–1^ (dark blue), 5 K·min^–1^ (green), 2 K·min^–1^ (red), and 1 K·min^–1^ (black).

**Figure 5:**
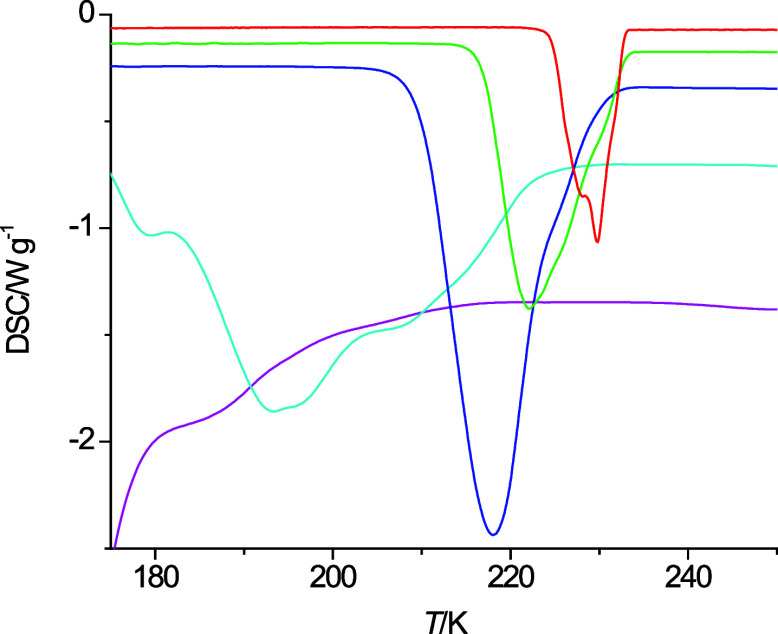
Cooling curves of 6-methoxyhexanol at 40 K·min^–1^ (pink), 20 K·min^–1^ (light blue), 10 K·min^–1^ (dark blue), 5 K·min^–1^ (green), and 2 K·min^–1^ (red).

Given that the molar masses are very similar for the ether alcohols in Table 1 compared to *n*-octanol, we initially anticipated freezing/melting to occur at similar temperatures as for *n*-octanol. As can be seen in Figure 1b–f, the ether alcohols do not show any evidence of freezing when cooled at a rate of 10 K·min^–1^ except for 6-methoxyhexanol in Figure 1f. 5-Ethoxypentanol shows an exothermic peak in the heating curve just below 200 K. This suggests that 5-ethoxypentanol was kinetically hindered from freezing during the cooling process and only during the heating process relaxed into the solid state before it melted again at a higher temperature, as evidenced by the endothermic peak near 225 K in the heating curve. Similar but less pronounced behavior is observed in Figure 1 for 2-pentoxyethanol and 4-propoxybutanol, as well as in Figure 3 for 6-methoxyhexanol at a temperature change rate of 40 K·min^–1^. The endothermic peak in the heating curves of 2-pentoxyethanol and 5-pentoxyethanol displays a splitting, which is indicative of two subsequent endothermic processes while these two ether alcohols transition from a solid phase to the liquid phase. 3-Butoxypropanol does not show any exo- or endothermic peaks even at the lowest investigated temperature change rate of 1 K·min^–1^ (not shown). The only tell-tale of some exothermic event happening during cooling process is a change in slope sign resulting in a very broad maximum near 225 K. Similar details are also observed for the cooling curves of 4-propoxybutanol (Figure 4) at a cooling rate of 40 K·min^–1^. Furthermore, the cooling curves of 6-methoxyhexanol shown in Figure 5 appear to transition with increasing cooling rate toward an absence of an exothermic freezing process.

**Figure 6:**
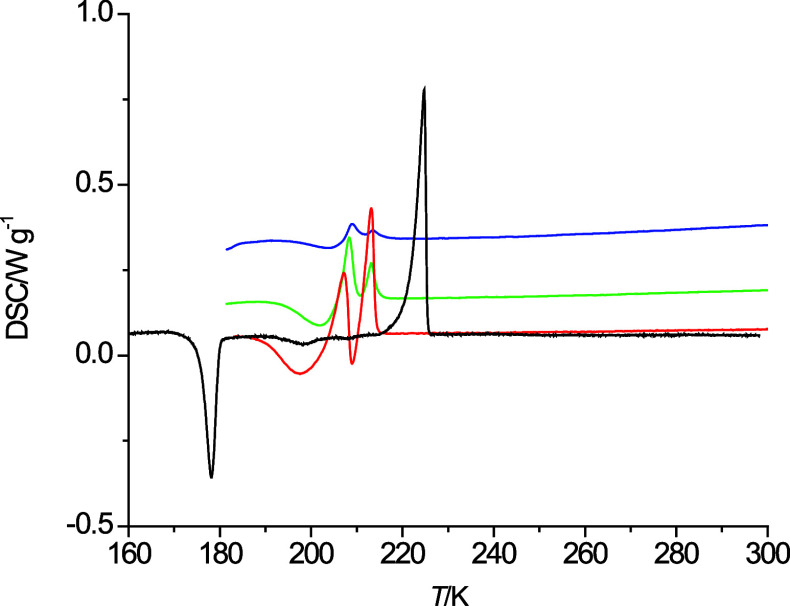
heating curves of 2-pentoxyethanol at 10 K·min^–1^ (dark blue), 5 K·min^–1^ (green), 2 K·min^–1^ (red), and 1 K·min^–1^ (black).

**Figure 7:**
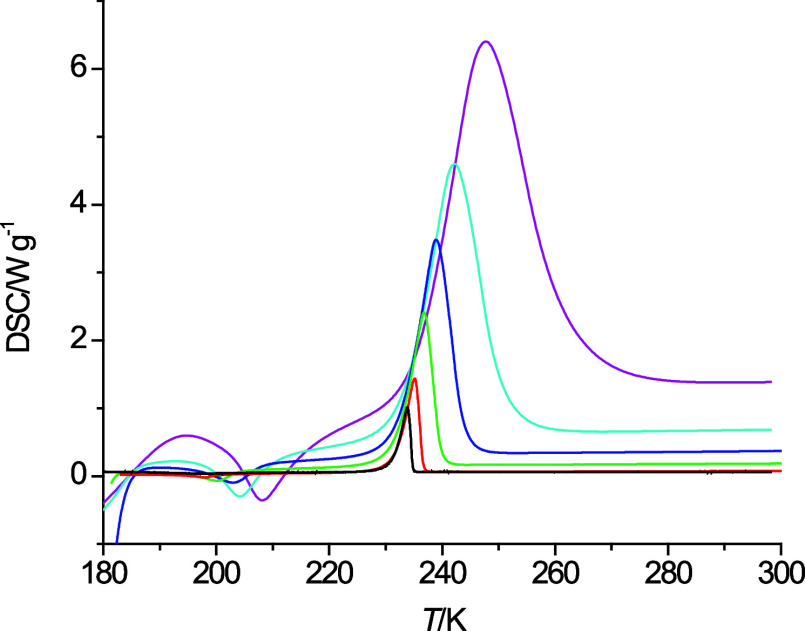
Heating curves of 4-propoxybutanol at 40 K·min^–1^ (pink), 20 K·min^–1^ (light blue), 10 K·min^–1^ (dark blue), 5 K·min^–1^ (green), 2 K·min^–1^ (red), and 1 K·min^–1^ (black).

**Figure 8:**
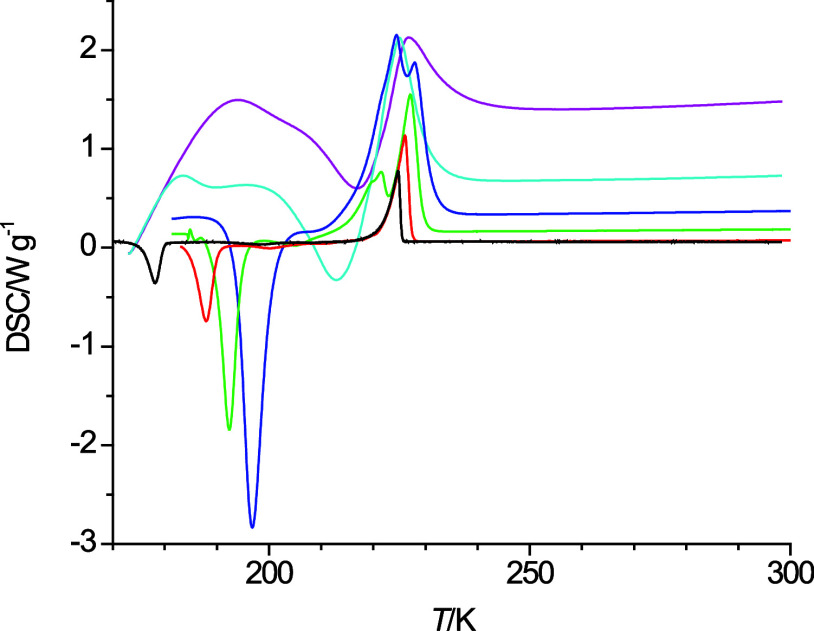
Heating curves of 5-ethoxypentanol at 40 K·min^–1^ (pink), 20 K·min^–1^ (light blue), 10 K·min^–1^ (dark blue), 5 K·min^–1^ (green), 2 K·min^–1^ (red), and 1 K·min^–1^ (black).

The heating curves at varying heating rates are shown in Figures 6–8 for 2-pentoxyethanol, 4-propoxybutanol, and 5-ethoxypentanol, respectively. For 2-pentoxyethanol, a clear separation of the exothermic freezing and endothermic melting processes is only observable for the lowest heating rate of 1 K·min^–1^. Thus, only one onset temperature for 2-pentoxyethanol could be read and was taken as the estimated melting point of 2-pentoxyethanol. For 4-propoxybutanol, the onset temperature is obscured at high temperature change rates by the exothermic feature from the freezing process and the melting temperature was estimated only from the heating curves with the lowest two heating rates. The same was done for 5-ethoxypentanol to obtain its melting point temperature as the traces obtained from the lowest heating rates do not show the splitting of the endothermic peak present at the higher heating rates. Table 2 summarizes the best estimates of the melting point temperatures, *T*_m_, of all investigated alcohols, except 3-butoxypropanol for which no clear phase transition was observed. Their associated standard uncertainties, *u*_*Tm*_, shown in Table 2 were estimated considering several sources of uncertainty discussed in the following. The agreement of the temperature of *n*-octanol with literature suggests an uncertainty contribution of 1 K from the temperature calibration of the DSC instrument. Additional uncertainty arises from the above-described difficulties in reading the onset temperature. Furthermore, as is well understood,^27^ the presence of impurities leads to a melting point depression, Δ*T*_m_, which is a colligative property calculable from the molality, *m*_2_, of the impurity according to eq 1
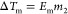
1where *E*_m_ is the cryoscopic constant, sometimes also referred to as the melting point depression constant. Values of *E*_m_ are not known for any of the studied ether alcohols. We are also unaware of any tabulated *E*_m_-values for *n*-octanol. However, from melting point depression data reported by Perlovich et al. *n*-Octanol appears to have an *E*_m_ value of 7 K·kg·mol^–1^.^11^ Also unknown is the molar mass of any of the present impurities, as the identity of the impurity is unknown. To gain insight into how large the melting point depression might be, we assume *E*_m_ to be similar to that of *n*-octanol and the molar mass of the impurity to be similar to that of the ether alcohol. The obtained melting point depression estimate from 5% mass fraction impurity is 2.8 K. The estimated combined uncertainties listed in Table 2 are the addition of the just described three uncertainty contributions (temperature calibration, reading onset temperature, and impurities) and thus vary case by case.

**Table 2:** Melting Points and Heats of Fusion and Their Combined Standard Uncertainties (*u*) of Studied Alcohols at Ambient Pressure (0.10 ± 0.01 MPa)

Alcohol	*T*_m_/K	*u_Tm_*/K	Δ*H*_f_/kJ mol^–1^	*u*_Δ*Hf*_/kJ mol^–1^
*n*-octanol	258	2	25.2	0.8
2-pentoxyethanol	219	7	15.4	2.8
4-propoxybutanol	231	4	18.0	0.9
5-ethoxypentanol	222	5	15.5	2.3
6-methoxyhexanol	250	4	18.7	1.9

### 3.2 Heats of Fusion

Table 2 also lists heats of fusion, Δ*H*_f_ associated with the listed melting points. These were obtained from the areas of the observed endothermic peaks in the respective heating curves. The heats of fusion are independent from the temperature change rate so that statistics can be obtained from the results obtained at varying temperature change rates. Results were scattered most for 2-pentoxyethanol and 5-ethoxypentanol likely due to the interfering effects on the baseline from the exothermic feature. We thus took the results obtained from the lowest measured heating rate as the most reliable value and estimate the standard uncertainty of these heats of fusion to be 2 kJ·mol^–1^. Standard deviations were also relatively high for the heats of fusion of 6-methoxyhexanol limiting its uncertainty to 1 kJ·mol^–1^. Standard deviations were well below the value of 0.5 kJ·mol^–1^ for 5-ethoxypentanol and *n*-octanol, but given the higher uncertainties observed for the other alcohols, we took that value as a conservative estimate. In addition, the uncertainties from present sample impurities need to be added to the uncertainties obtained from the standard deviations of repeated measurements. Table 2 lists these combined standard uncertainties, which are rather conservative estimates amounting to up to 18% relative uncertainty for 2-pentoxyethanol. van Miltenburg et al. reported a value of 25.24 kJ·mol^–1^ for the heat of fusion of *n*-octanol,^9^ which is in very good agreement with the value in Table 2 obtained from this study. Perlovich et al. reported a somewhat lower value of 22.46 kJ·mol^–1^.^11^

### 3.3 Isobaric Molar Heat Capacities

**Figure 9:**
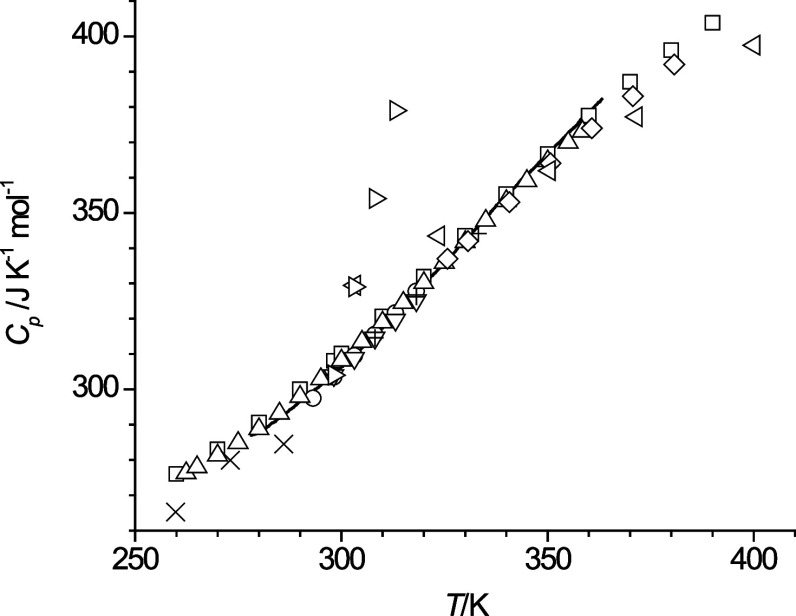
Molar isobaric heat capacity (*C*_p_) of *n*-octanol as a function of temperature, comparing results from this study (solid line) with data from van Miltenburg et al.^9^ (squares), Dzida^28^ (circles), Pokorný et al.^29^ (triangle-up), Vesely et al.^30^ (triangle-down), and Fulem et al.^12^ (diamonds), Cline and Andrews^21^ (cross), Páramo et al.^18^ (plus), Naziev et al.^20^ (triangle-left), Anouti et al.^19^ (triangle right).

The slopes of the heating and cooling curves in temperature ranges outside of any phase transitions are proportional to the isobaric molar heat capacity, *C*_p_. While slower temperature change rates provide more resolution of the heating and cooling curves, they are also becoming noisier. From preliminary calibration measurements with sapphire,^25^ a rate of 10 K·min^–1^ appeared to be the best compromise between these limiting factors to result in the most reliable heat capacity data. Isobaric molar heat capacities are only reported for the liquid phase given the observed uncertain phase behavior of the ether octanols below their respective melting points. Isobaric molar heat capacity data over similar temperature ranges investigated here are reported in the literature for *n*-octanol and are shown with the *C*_p_ data obtained in this report in Figure 9. The heat capacity data of this study are shown in Figure 9 as a line because they were recorded in increments smaller than 0.1 K so that showing these as data points would anyway have optically the appearance of a line. Most of the data sets shown in Figure 9 optically coincide. The data sets that clearly deviate from the majority of the data are the three data points reported by Cline and Andrews,^21^ who measured these values by a thermal conductivity method, and by Anouti et al.,^19^ whose *C*_p_ data show a much steeper temperature dependence than the other data sets. Conversely, the *C*_p_ data reported by Naziev et al.^20^ display a significantly flatter temperature dependence than the other data sets. From the remaining data sets in Figure 9, the largest deviation of 3.8 J·K^–1^·mol^–1^ to the *C*_p_ values of *n*-octanol reported here is observed for the data from van Miltenburg et al. at 300 K,^9^ which amounts to a relative deviation of 1.24%. The *C*_p_ values at 300 K reported by Dzida^28^ and Pokorný et al.^29^ are in better agreement with the *C_p_* values from this study, within 1.8. J·K^–1^·mol^–1^, and the data sets in Figure 9 from Vesely et al.^30^ and Páramo et al.^18^ agree to within 0.4 J·K^–1^·mol^–1^ at 298.15 K. Additional single data points for the heat capacities of *n*-octanol at 298.15 K are reported by Domalski and Hearing^14^ (305.55 J·K^–1^·mol^–1^), Rubini et al.^15^ (304.4 J·K^–1^·mol^–1^), Calvo et al.^17^ (305.3 J·K^–1^·mol^–1^), and Naef^16^ (312.1 J·K^–1^·mol^–1^). In contrast to the value by Naef, the *C*_p_ values by Calvo et al. and Domalski and Hearing are in line and the value by Rubini even in complete agreement with our obtained *C*_p_-value at 298.15 K. Also included in Figure 9 is a data set reported by Fulem et al.,^12^ which has been acquired at a higher than ambient pressure of 2 MPa. These reported values are lower than the values of this study by up to 6.1 J·K^–1^·mol^–1^ where the deviations increase with temperature. The data set by Pokorný et al.^29^ in Figure 9 also shows smaller values at the highest temperature, but only deviating up to 3.6 J·K^–1^·mol^–1^. However, the reported data by Fulem et al.^12^ as well as by Naziev et al.^20^ show that increasing pressure decreases *C*_p_, which implies that their values would deviate even more from the values reported by Pokorný et al. and reported in this study if they had been measured at ambient pressure. Overall, from the comparison with the literature data, a value of 3 J·K^–1^·mol^–1^ appears to be a conservative estimate for the standard uncertainty of the *C*_p_ values reported in this study.

As can be seen in Figure 1, the heating and cooling curves from the liquid phases are nearly linear, which means that heat capacities show rather small deviations from a linear temperature dependence. This can indeed be seen for *n*-octanol in Figure 9. The *n*-octanol data in Figure 9 were fitted to a third order polynomial with respect to temperature resulting in a fit line that is indistinguishable from the data line shown in Figure 9. Deviations from linearity appeared to be even less for the *C*_p_ data of the ether alcohols such that fitting with a second order polynomial resulted in better fit statistics than fitting with a third order polynomial. Table 3 summarizes the fit coefficients along with the temperature ranges and the standard deviation of the fit to the data. These standard deviations of the fit to the *C*_p_ data are smaller than the estimated standard uncertainty of 3 J·K^–1^·mol^–1^. Table 4 reports the interpolated *C*_p_ values at 5 K increments obtained from applying the polynomial fit equations of Table 3.

**Table 3:** Polynomial Fitting Coefficients for the Temperature Dependent Isobaric (0.10 ± 0.01 MPa) Molar Heat Capacities in J·K^–1^·mol^–1^ (*C*_p_ = *a*_3_*T*^3^ + *a*_2_*T*^2^ + *a*_1_*T* + *a*_0_)

Alcohol	*a*_3_/10^–5^	*a*_2_/100	*a*_1_	*a*_0_	*T*_min_/K	*T*_*max*_/K	*σ*_*fit*_^a^/J K^–1^ mol^–1^
*n*-octanol	–6.6164	6.5765	–20.5279	2332.3	278.15	363.15	0.2
2-pentoxyethanol	0	0.1125	–0.10152	232.6	243.15	373.15	0.6
3-butoxypropanol	0	0.1083	–0.03484	214.4	243.15	383.15	0.7
4-propoxybutanol	0	0.2086	–0.64212	298.3	253.15	363.15	0.3
5-ethoxypentanol	0	0.2377	–0.80762	323.8	243.15	353.15	0.7
6-methoxyhexanol	0	0.2586	–0.95978	352.3	273.15	353.15	0.3

a *σ*_fit_ is the standard deviation of the fit to the data.

**Table 4:** Isobaric (0.10 ± 0.01 MPa) Molar Heat Capacities^a^

	*n^b^*					
*T*/K	0	2	3	4	5	6
243.15		274.4	269.9		267.9	
248.15		276.6	272.4		269.7	
253.15		279.0	275.0	269.4	271.7	
258.15		281.3	277.6	271.6	273.7	
263.15		283.8	280.2	273.8	275.9	
268.15		286.2	282.9	276.1	278.1	
273.15		288.8	285.7	278.6	280.5	283.1
278.15	286.7	291.4	288.5	281.1	283.0	285.4
283.15	290.4	294.0	291.3	283.7	285.7	287.9
288.15	294.6	296.7	294.3	286.5	288.4	290.5
293.15	299.3	299.5	297.2	289.3	291.3	293.2
298.15	304.4	302.3	300.3	292.3	294.3	296.0
303.15	309.7	305.2	303.4	295.4	297.4	299.0
308.15	315.4	308.1	306.5	298.5	300.6	302.1
313.15	321.3	311.1	309.7	301.8	304.0	305.3
318.15	327.3	314.1	312.9	305.2	307.4	308.7
323.15	333.5	317.2	316.2	308.6	311.0	312.2
328.15	339.8	320.4	319.6	312.2	314.7	315.8
333.15	346.1	323.6	323.0	315.9	318.6	319.6
338.15	352.4	326.9	326.4	319.7	322.5	323.4
343.15	358.6	330.2	330.0	323.6	326.6	327.5
348.15	364.7	333.6	333.5	327.6	330.7	331.6
353.15	370.6	337.0	337.2	331.7	335.0	335.9
358.15	376.4	340.5	340.8	335.9		
363.15	381.8	344.0	344.6	340.2		
368.15		347.6	348.4			
373.15		351.3	352.2			
378.15			356.1			
383.15			360.0			

a Estimated standard uncertainty is 3 J·K^–1^·mol^–1^ with estimated standard uncertainty of 1 K for the temperature readings.

b *n* refers to the alcohols with *n* = 0 for *n*-octanol, *n* = 2 for 2-pentoxyethanol, *n* = 3 for 3-butoxypropanol and so on.

In summary, the observed thermal behavior of the ether alcohols is clearly different from that of *n*-octanol. There is significant differentiation between the individual ether octanols, with 6-methoxyhexanol and, to a lesser extent, 4-propoxybutanol showing the strongest tendency for the usual phase behavior of forming a crystalline solid upon cooling while the other ether alcohols are not forming a solid crystalline phase upon cooling especially 3-butoxy-1-propanol. It is apparent that the increased hydrogen bonding possibilities coming about with the ether functionality generally makes it harder for the molecules to align into a crystal structure. In the case of 6-methoxyhexanol, intramolecular hydrogen bonding should contribute the least to the overall hydrogen bonding interactions compared to the other ether alcohols. This may allow 6-methoxyhexanol more easily to form a crystalline solid compared to the other ether alcohols. Conversely, intramolecular hydrogen bonding appeared to be most prevalent for 3-propoxybutanol and 4-propoxy-butanol based on prior density, viscosity and self-diffusion measurements.^1^ Interestingly, while 3-propoxybutanol does not form a crystalline solid at all in this DSC study, 4-propoxybutanol displayed the second strongest propensity to form a crystalline solid after 6-methoxyhexane. Possibly, the structural conformations induced by intramolecular hydrogen bonds may allow for a structural ordering unique to 4-propoxybutanol that facilitates the formation of a solid crystalline phase upon cooling. More experimental and theoretical studies such as, for example, X-ray diffraction and molecular dynamics (MD) studies that are beyond the scope of this report are needed to test this hypothesis.

## 4 Conclusion

New data on melting point, heats of fusion and isobaric heat capacities on *n*-octanol related ether alcohols have been reported. In contrast to *n*-octanol, the presence of the ether functional group in the ether alcohols introduces barriers for the formation of a crystalline solid, which is most pronounced for 3-butoxypropanol that does not show any indication of a liquid-to-solid phase transition. The other ether alcohols tend to undergo to varying degrees a liquid-to-solid phase transition during the heating cycle of the DSC experiment. The details of this behavior are strongly dependent on the temperature change rates where 6-methoxyhexanol only displays this behavior at very high temperature change rates. Underlying this varied phase behavior is likely the interplay of inter- and intramolecular hydrogen bonding between hydroxy hydrogen and ether oxygen in addition to intermolecular hydrogen bonding between hydroxy groups, where the latter is the only hydrogen bonding interaction present in *n*-octanol. Further studies are needed to unravel these hydrogen bonding structures and dynamics and explain on a molecular level the varied thermal behavior of the ether alcohols observed in this study.
